# Autophagy in Cancer Cell Death

**DOI:** 10.3390/biology8040082

**Published:** 2019-10-29

**Authors:** Benedikt Linder, Donat Kögel

**Affiliations:** 1 Experimental Neurosurgery, Department of Neurosurgery, Neuroscience Center, Goethe University Hospital, 60528 Frankfurt am Main, Germany; 2 German Cancer Consortium (DKTK), Partner Site Frankfurt, 60590 Frankfurt am Main, Germany

**Keywords:** autophagy, cell death, cancer, gossypol, AT-101, mitophagy, mitochondria

## Abstract

Autophagy has important functions in maintaining energy metabolism under conditions of starvation and to alleviate stress by removal of damaged and potentially harmful cellular components. Therefore, autophagy represents a pro-survival stress response in the majority of cases. However, the role of autophagy in cell survival and cell death decisions is highly dependent on its extent, duration, and on the respective cellular context. An alternative pro-death function of autophagy has been consistently observed in different settings, in particular, in developmental cell death of lower organisms and in drug-induced cancer cell death. This cell death is referred to as autophagic cell death (ACD) or autophagy-dependent cell death (ADCD), a type of cellular demise that may act as a backup cell death program in apoptosis-deficient tumors. This pro-death function of autophagy may be exerted either via non-selective bulk autophagy or excessive (lethal) removal of mitochondria via selective mitophagy, opening new avenues for the therapeutic exploitation of autophagy/mitophagy in cancer treatment.

## 1. Introduction

### 1.1. Programmed Cell Death 

In order to remove damaged or unwanted cells, multicellular organisms rely on multiple forms of programmed cell death (PCD). PCD is an evolutionarily-conserved phenomenon and the most common and best-studied form of PCD is apoptosis (type I cell death) [1]. However, multiple alternative cell death programs have been described in the last decades [2,3]. Apoptotic cell death is believed to be the main mode-of-action of most conventional chemotherapeutics and radiation therapy in tumor treatment. Much effort has been invested into deciphering the components of the cellular apoptotic machinery and several targeted agents have already been developed to specifically trigger apoptosis in an aim to better combat cancer in the clinic. However, many cancers are highly resistant to apoptosis and numerous studies illustrate that alternative, non-apoptotic forms of PCD [2,4] exist, some of which might represent suitable therapeutic avenues, in particular, for the treatment of apoptosis-refractory cancers.

### 1.2. Autophagy in Cell Survival and Cell Death

**Figure 1:**
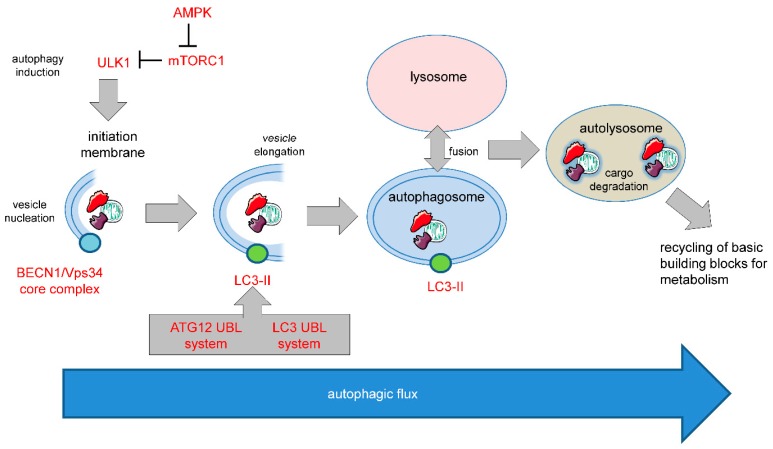
Molecular basis of mammalian autophagy. Autophagy is a multistep process involving several key ATG proteins and signaling complexes. It requires the formation of double-membrane-containing autophagosomes that sequester proteins, lipids, organelles or invasive microbes and fuse with lysosomes for digestion of content by acidic hydrolases. ULK1, a protein kinase serving as the central initiator of autophagy, is inhibited by the mTORC1 complex that contains mTOR. AMPK serves as a nutrient sensor and negative regulator of mTORC1. Autophagosome biogenesis starts with the formation of an initiation membrane that is derived either from the endoplasmatic reticulum (ER) or from several other cellular membrane sources. Vesicle nucleation is promoted by the BECN1/Vps34 core complex containing the lipid kinase Vps34. Vesicle elongation is regulated by the two ubiquitin-like conjugation systems (UBLs) ATG12-UBL and LC3-UBL that cooperate to catalyze the conjugation of phosphatidylethanolamine (PE) to LC3 and facilitate the conversion of cytosolic LC3-I into a membrane-associated LC3-II that is translocated to the autophagosomal membrane. Following vesicle closure, mature autophagosomes fuse with lysosomes to generate autolysosomes that digest the autophagosomal content by lysosomal proteases for cellular recycling [5]. This figure was created using Servier Medical Art templates, which are licensed under a Creative Commons Attribution 3.0 Unported License; https://smart.servier.com.

One such apoptosis-independent cell death mechanism relies on the over activation of autophagy, a cellular stress response that normally serves as a quality control mechanism. Different forms of autophagy have been described including macroautophagy (hereafter referred to as autophagy), microautophagy, and chaperone-mediated autophagy. The molecular basis of mammalian autophagy is depicted in Figure 1.

**Figure 2:**
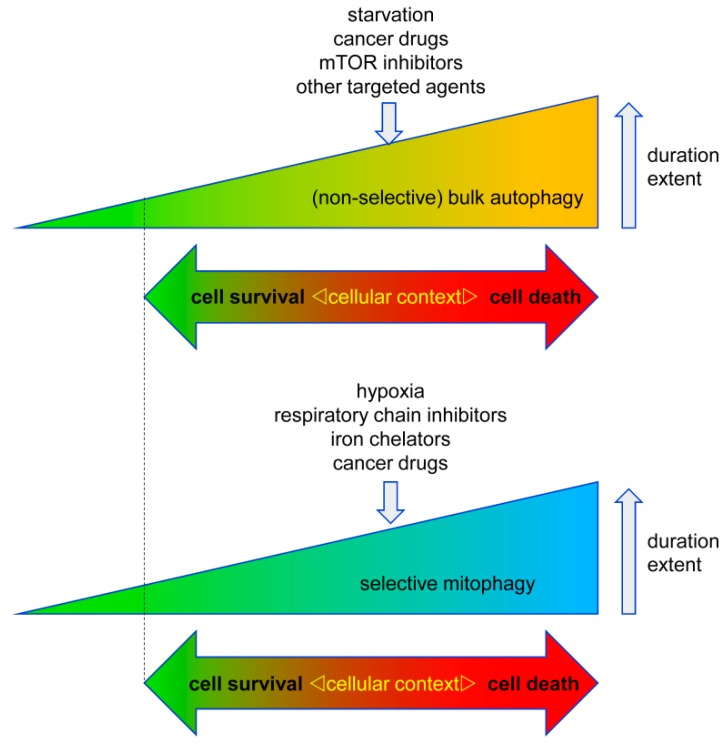
Context-dependent cell responses to bulk autophagy and selective mitophagy. Both autophagy and mitophagy can either promote or inhibit cell death in cancer cells. This response is highly dependent on the cell type, the trigger of auto-/mitophagy, its duration, and its extent. Accordingly, excessive autophagy can lead to cell death, however too little autophagy/mitophagy (marked by dotted line) can also be detrimental to the cells due to an impaired quality control/removal of harmful cellular material.

In general, autophagy is a pro-survival stress response, for example, autophagy will be activated under situations of nutrient deprivation to ensure supply of basic building blocks for metabolism and survival of the cells/organisms by recycling of non-essential cellular components. Autophagy also serves to remove damaged and potentially harmful organelles, thereby supporting cell survival. On the other hand, there is conclusive evidence that prolonged over activation of the autophagosomal/lysosomal pathway can lead to autophagic cell death (ACD, type II cell death). Of note, similar threshold effects on cell survival vs cell death are commonly observed in various stress responses like the endoplasmatic reticulum (ER) stress response and activation of p53 [6]. Accordingly, ACD is often described as self-digestion beyond the point allowing cell survival [3,4,6,7,8]. Hence, the net effect of autophagy on cell survival is highly dependent on its intensity and duration, but also on its particular context (Figure 2). 

Recently, ACD was further defined by the Nomenclature Committee on Cell Death (NCCD) based on molecular/functional parameters [3]. Specifically, the term ACD shall only be used in cases where cell death is genetically linked to the core autophagy pathway (analyzed either by Vps34 inhibitors or by knockout/knockdown of core autophagy factors like ATG5, ATG12 or BECN1) and when cell demise does not engage other forms of PCD [3,9]. Notably, some authors even suggested more stringent criteria, implying that the final cell death process is functionally linked to an enhanced autophagic flux [5,10], although this flux will ultimately have to break down at some point in dying/dead cells. 

Although the most convincing observations of ACD have been made using lower model organisms like *Caenorhabditis elegans*, *Drosophila melanogaster*, and *Dyctiostelium discoideum* [5,11,12,13], numerous studies have also demonstrated bona fide ACD in mammalian cells [14,15,16,17,18,19]. Liu et al. observed that a cell-permeable Tat-Beclin 1 peptide induces a (bulk) autophagy-dependent type of cell death that could be inhibited with antagonists of Na(+), K(+)-ATPase and the authors termed this type of cell death as “autosis” [20]. Whether autosis is just another variation of ACD or may indeed represent a different cell death entity is currently under debate. 

## 2. Autophagy in Tumorigenesis and Tumor Progression

Autophagy is not only involved in the responses to tumor therapy (see below), but also plays a key role in cancer development. This involvement of autophagy in tumorigenesis and tumor progression is very complex and multifaceted. It is hypothesized that in the early stages of tumorigenesis, autophagy exerts a tumor-suppressive function. This is based on the observation that an intact autophagy pathway correlates with decreased oxidative stress and increased genomic stability [8], thereby ensuring the survival of healthy, non-transformed cells. On the other hand, it was recently proposed that autophagy may also support cancer progression by facilitating tumor cell survival and fitness under replication stress, a common feature of most malignancies [21]. Adding a further layer of complexity, Nassour et al. recently demonstrated that ACD can serve as a final barrier for oncogenic transformation during replicative crisis. In this setting, autophagy-inhibition resulted in continuous growth and accumulation of genomic instability [22]. In addition, key components of the autophagic machinery, like AMBRA1 (autophagy and beclin1 regulator 1) and BECN1 (Beclin1) [23,24], are considered to be tumor-suppressors. Specifically, BECN1 is monoallelically deleted in several types of cancer, including breast, ovarian, and prostate cancer [23]. Its interaction partner, UV irradiation resistance-associated gene (UVRAG), frequently exhibits monoallelic mutations in human colon cancers [25]. Another BECN1-binding protein, Bif-1 (also known as Endophilin B1), can display tumor-suppressive functions since Bif-1 knockout leads to spontaneous tumor development in mice [26]. AMBRA1-deficiency leads to uncontrolled cell proliferation as well, and AMBRA1 can also interact with BECN1 [27], again supporting a tumor-suppressive function of autophagy. 

In line with the high context-dependency of autophagy, there is a shift from tumor-suppressing to tumor-promoting autophagy during the course of tumor progression. It is believed that autophagy can alleviate the stressful environmental conditions like hypoxia or nutrient deprivation often encountered in manifest, solid tumors [28,29], but also in non-malignant ischemic tissues [30], rendering the tumors more stress-resistant. Using a *Drosophila* model, Katheder et al. recently showed that not only tumor-intrinsic but also microenvironmental autophagy is capable of inducing tumor growth by providing the nutrients necessary for tumor growth [31]. They could further demonstrate that this was achieved through elevated ROS levels due to mitochondrial damage in the tumor cells, which induced nutrient export from the microenvironment. 

Since another key hallmark of cancer is chronic inflammation, it will also be of key importance to better understand the mutual interplay between autophagy and inflammation. There is evidence suggesting that autophagy can either suppress or promote inflammation in cancer. Likewise, inflammatory pathways can either suppress or induce autophagy in a context-dependent manner. A complex scenario is recently emerging that will aid future studies aimed at deciphering the exact role of autophagy in shaping the immune and inflammatory microenvironment of tumors [32].

In addition to supporting tumor growth in general, autophagy has been demonstrated to regulate and/or maintain the cancer stem cell phenotype and treatment resistance in multiple studies, for example, in oral squamous cell carcinoma [33] and endometrial cancer [34].

## 3. Autophagy in Therapy Response

Next to its role in tumorigenesis and malignant progression, autophagy plays a key role in cancer therapy responses. Given the dual function of autophagy in cell survival vs cell death, inhibition, but also over activation of autophagy, carries potential relevance for therapy. 

### 3.1. Pro-Survival Autophagy

Since autophagy appears to act mainly as a pro-survival stress response that is activated (at least to some degree) by most, if not all, conventional cancer drugs and by radiation, pro-survival autophagy is expected to hamper the effects of cancer therapy in most settings. Some examples of a therapy resistance-increasing effect of autophagy are listed below. The impact of pro-survival autophagy in cancer therapy was extensively covered elsewhere in our recent review [35] where we also delineated the molecular mechanisms of autophagy regulation in response to therapy-related stress conditions in this context, and we refer the reader to this work for further details. Two central cellular players involved in many paradigms of pro-survival autophagy of cancer cells are mTOR and AMPK (Figure 1) that are often involved in activation of autophagy as an unwanted side effect of different cancer drugs/treatments. For example, treatment with Taxol was shown to activate pro-survival autophagy caused by inhibition of mTOR in breast cancer cells [36]. Accordingly, activation of AMPK, the endogenous negative regulator of mTOR, using Bortezomib has been reported to induce pro-survival autophagy in pancreatic and colorectal cancer cells [37]. In addition to the net effect of (pro-survival) autophagy inhibition on cell death, there is crosstalk between autophagy and apoptosis. For example, the transcription factor FOXO3a is degraded by basal autophagy and increased FOXO3a levels upon autophagy inhibition stimulate the induction of the pro-apoptotic BBC3/PUMA gene, thereby sensitizing cells to apoptosis-inducing chemotherapeutics [38]. However, autophagy can also promote apoptosis in some cases. Another study from the same group demonstrated that selective autophagic degradation of the phosphatase Fap-1 promotes Fas apoptosis in type I cells that do not require mitochondrial permeabilization for efficient apoptosis, while autophagy inhibited apoptosis in type II cells [39]. Additional mechanisms/molecular players involved in pro-survival autophagy are briefly listed below. 

For pancreatic cancer, it has been shown that primary tumors and cell lines exhibit increased autophagy, while autophagy inhibition (genetic and pharmacological) results in increased reactive oxygen species (ROS) formation and DNA damage, while treatment of tumor-bearing mice with the autophagic flux-inhibitor chloroquine (CQ) improved overall survival [40]. In another study Qiu et al. showed that autophagy induced by cisplatin protected ovarian cancer cells [41], while DeVorkin et al. could, in fact, show that cancer cells of clear-cell ovarian cancer depend on autophagy for their survival [42]. From a mechanistic perspective, it should, however, be noted that CQ is not a highly selective inhibitor of autophagy. A recent study demonstrated that CQ also has profound non-autophagic effects on cells, especially concerning disorganization of the Golgi and endo-lysosomal systems [43], arguing for a more cautious interpretation of responses to CQ.

The approach of combining conventional or targeted therapy with autophagy inhibition (CQ, Hydroxy-CQ) is currently also investigated in several clinical studies in patients with various types of cancer, including glioblastoma. Accordingly, Jutten et al. showed recently that glioma cells expressing mutant EGFRvIII that is associated with poor prognosis [44] and occurs in half of all glioblastoma patients [45], are more sensitive to CQ treatment, and hence rely more strongly on autophagy for cell survival. Most importantly, using a retrospective analysis, this study also showed that patients with mutant EGFRvIII receiving CQ have the highest benefit of CQ-treated patients [46]. Another recent, very promising study provided evidence that autophagy inhibition can be employed to overcome therapy resistance of brain tumor patients against BRAF inhibitor treatment [47]. 

### 3.2. Pro-Death Autophagy

Given the fact that genetic and pharmacological abrogation of autophagy inhibits non-selective as well as selective types of autophagy, it is currently not well understood whether excessive pro-death bulk autophagy, i.e., non-selective autophagy, is the (solely) responsible type of autophagy for cell killing in most established paradigms of ACD, including ACD in lower organisms and ACD induced by cancer drugs. The following section lists several examples from the literature that lack evidence for a death-promoting contribution of selective autophagy pathways, such as mitophagy (see next paragraph).

Resveratrol, a polyphenolic compound found in red wine [48], has been described to induce bona fide ACD in chronic myeloid leukemia [15] and induces cell death in prostate [49], ovarian [16], and endometrial cancer cells [50] that involves induction of autophagy, although the latter studies failed to provide complete evidence that the criteria required by the NCCD [3] are fulfilled. A recent shRNA-based screen of A549 lung cancer cells analyzed potential regulators of resveratrol-induced ACD and identified glucosylceramidase beta (GBA1) as a potential mediator of ACD [51]. ACD has also been observed in cells treated with Interferon-gamma (IFN-γ) which induced cell death that could be rescued after treatment with the autophagy-inhibitor 3-methyl-adenine (3MA) or knockdown of ATG5 [17]. Based on the observations that cancer cells have a higher turnover rate of NAD+, this pathway was recently employed to target cancer cells by triggering ACD via inhibition of the NAD+-synthesizing enzyme Nampt using the inhibitor FK866 in myeloma [52] or by inhibition of the nicotinamide phosphoribosyltransferase by APO866 in leukemia and lymphoma cells [53]. Lima et al. used SK1-I, an inhibitor of sphingosine kinase 1 (SPHK1) and analog of sphingosine, in colon cancer cell lines and observed induction of autophagy and cell death which was dependent on BECN1 and ATG5 [54], although in this study the discrimination between apoptosis and autophagy is not entirely clear, leaving some room for interpretation if the mode of death can be truly defined as ACD according to the NCCD criteria. Other groups showed that downregulation of the AKT1/mTOR-axis using the histone deacetylase inhibitor suberoylanilide hydroxamic acid (SAHA) induced ACD in hepatocellular carcinoma (HCC) cell lines [55]. Finally, the cholesterol metabolite dendrogenin A (DDA) induced lethal autophagy, reminiscent of ACD, in myeloma and acute myeloid leukemia in vitro and in vivo [19]. 

Arsenic trioxide was shown to induce ACD and cell death in various tumor cell populations in multiple studies, including our own [14,56,57,58]. Considering that arsenic trioxide is already clinically used to treat acute promyelocytic leukemia (APL) [59] and easily crosses the blood-brain-barrier [60], this drug could be particularly interesting for hard-to-treat cancers, such as brain tumors (primary or metastases). In particular, it was shown that arsenic trioxide-induced ACD is mediated by the protein BNIP3 (BCL2 interacting protein 3) and BNIP3L (BCL2 interacting protein 3 like; also known as NIX) [14], that were subsequently identified as mitophagy-receptors [61]. These findings imply that arsenic trioxide triggers selective autophagy of mitochondria (mitophagy) in addition to non-selective bulk-autophagy, with possible implications for cell death activation. However, this proposition warrants future research.

An alternative approach to trigger ACD with autophagy-inducing drugs is the use of different metallic nanoparticles to overstimulate autophagy in an aim to induce ACD in cancer cells. This approach was recently reviewed elsewhere and we would like to refer the reader to this work [62].

#### 3.2.1. Pro-Death Selective Autophagy

**Figure 3:**
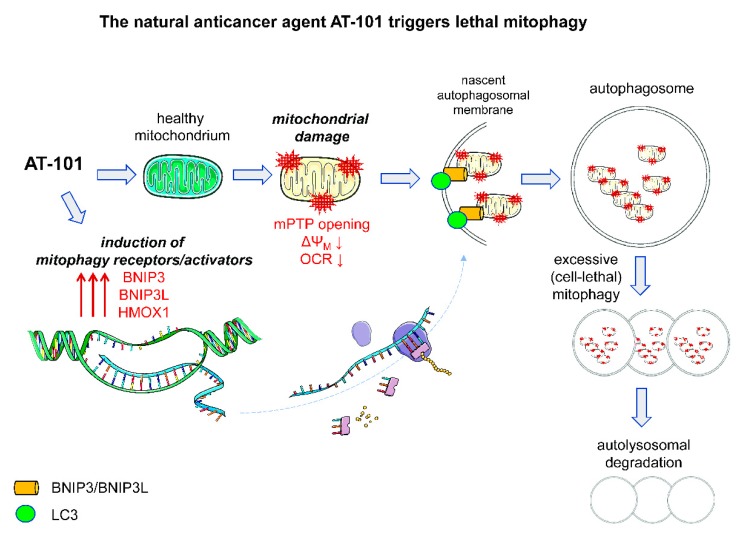
AT-101/gossypol as a trigger of lethal mitophagy. AT-101 causes mitochondrial damage including mitochondrial permeability transition pore (mPTP) opening, loss of membrane potentials, and decreased oxygen consumption. Damaged mitochondria are selectively degraded via mitophagy. In parallel, AT-101 induces the mitophagy receptors BNIP3 and BNIP3L/NIX, as well as the mitophagy-inducer HMOX1, which will further facilitate the extent of mitophagy. This excessive mitophagy finally leads to the demise of the cancer cell(s). This figure was created using Servier Medical Art templates, which are licensed under a Creative Commons Attribution 3.0 Unported License; https://smart.servier.com.

The role of selective autophagy pathways in promoting ACD is not well established and has been exclusively studied for the pro-death function of (excessive) mitophagy so far (Figure 3). It is currently unclear whether other types of selective autophagy such as ER-phagy or pexophagy may also actively contribute to cell death in some settings and future studies will hopefully address this aspect in detail.

One example of lethal mitophagy was demonstrated by Wang et al. who showed that 1-(3,4,5-trihydroxyphenyl) nonan-1-one, a compound targeting the orphan nuclear receptor TR3/Nur77, induces ACD by autophagy-dependent excessive removal of mitochondria and induction of cell death [63]. Other studies revealed that C-18 ceramide [64] and the mitochondria-targeted ceramide analog LCL-461 [65] induced lethal mitophagy in head and neck squamous cell carcinoma and FLT3-ITD (Fms-like tyrosine kinase 3-internal tandem repeat)-positive acute myeloid leukemia (AML) cells, respectively. Similarly, selenite was suggested to cause lethal mitophagy in glioma cells [66,67]. 

#### 3.2.2. Pro-Death Mitophagy Triggered by Gossypol/AT-101

Gossypol is derived from cotton seeds (genus *Gossypium*) and was initially tested as a male contraceptive agent in China, Africa, and Brazil [68]. Due to cases of irreversible infertility in up to 20% of men, this use was discontinued. Later on, gossypol was identified as a BH3 mimetic, a drug that can inhibit anti-apoptotic members of the BCL-2 family, and was shown to evoke cell death in multiple *in vivo* and *in vitro* models [69,70,71,72,73,74]. Gossypol can occur as two racemic enantiomers, (+)-gossypol and (−)-gossypol (also called AT-101), with AT-101 being more potent as a cancer drug. AT-101 has two main modes of inducing cell death. In apoptosis-proficient cancer cells, it induces apoptotic cell death [75,76,77,78,79]. However, in apoptosis-deficient cancer cells like prostate cancer and malignant glioma, AT-101 triggers ACD [73,80].

Recently, we established human MZ-54 GBM cells with CRISPR/Cas9-mediated KO of ATG5 or ATG7 to test the relevance of autophagy for drug-induced signaling and cell death. We discovered that AT-101 specifically induces ACD in GBM via a mitophagic type of cell death. Our analysis revealed a key role of mitochondrial events in AT-101-stimulated cell death. In particular, we found that AT-101 impaired mitochondrial respiration and rapidly triggered mitochondrial membrane depolarization. We also observed the engulfment of mitochondria within autophagosomes using electron microscopy (EM), and a significant reduction of mitochondrial mass and proteins, as determined with the mito-Keima assay and by global proteomic analysis of U87 and U343 GBM cells, that neither depend on the presence of Parkin nor the proapoptotic BCL-2 family proteins BAX and BAK1 [73,81,82].

Importantly, AT-101-induced cell death was significantly rescued in ATG5 or ATG7 KO GBM cells. Additionally, AT-101-induced reduction of mitochondrial mass could be reversed by pharmacological inhibition of autophagy. Silencing of heme oxygenase 1 (HMOX1) and the mitophagy receptors BCL2-interacting protein 3 (BNIP3) and BNIP3-like (BNIP3L), significantly attenuated AT-101-dependent mitophagy and cell death. Collectively, these data suggest that early mitochondrial dysfunction and HMOX1 over activation synergize to trigger lethal mitophagy following AT-101 treatment of GBM cells (Figure 3) [81,82].

As mentioned above, AT-101 treatment resulted in a robust upregulation of HMOX1 that is best known for its role in the degradation of pro-oxidant heme but was also shown to be important for mitochondrial biogenesis and mitophagy [83,84]. Specifically, doxorubicin-treatment of cardiac myocytes induced HMOX1 expression, which in turn led to the upregulation of NFE2L2 (nuclear factor, erythroid 2 like 2), PPARGC1A (PPARG coactivator 1 alpha), and TFAM (transcription factor A, mitochondrial) [85]. Nonetheless, as also described by others, HMOX1 can act in a cytoprotective or cytotoxic manner via induction of autophagy [83]. Infection-induced sepsis could be ameliorated in hepatocytes by HMOX1-dependent upregulation of autophagy [86]. A cardiomyocyte-specific knockout of HMOX1 results in disturbed mitochondrial quality control [87]. However, in a model of neurodegeneration, HMOX1 overexpression activates mitophagy and leads to cell death [88], and in macrophages, HMOX1 induces oxidative stress and mitochondrial dysfunction resulting in increased autophagy [89]. Using our model of AT-101-induced, mitophagy-mediated cell death, we could diminish the cell killing of AT-101 which was accompanied by decreased mitophagy after HMOX1-knockdown [81]. 

Interestingly, we could also recently show that the combination of arsenic trioxide and AT-101 caused a strong upregulation of HMOX1 in glioma stem-like cells (GSCs) [56], indicating that more specialized cell populations can also be targeted with drugs employing these pathways. In summary, we recently provided novel evidence that the decrease in mitochondrial mass and function induced by AT-101 is due to robust over activation of mitophagy that finally culminates in the demise of the cancer cells.

## 4. Outlook

The dual function of autophagy acting as a tumor suppressor or tumor promoter is well established and highly dependant on the exact context and the extent of autophagy engagement. Many cancer drugs, but also “physiological” processes within cancers like hypoxia or nutrient shortages, trigger autophagy. Hence, it is of paramount importance to better understand tumor- specific requirements for autophagy. This can finally lead to rationalized exploitation of autophagy-inhibition or further activation of it to increase the therapeutic response. Similarly, the induction of selective autophagy like mitophagy can negatively and positively affect survival, depending on the context and extent, for example, by preventing ROS-formation due to damaged mitochondria (pro-survival) or by removing the major energy source of the cells (pro-death). Future research should therefore focus especially on discriminating between bulk autophagy and selective types of autophagy such as mitophagy, and by defining the molecular switches regulating these processes. This is especially important since it is conceivable that other forms of selective autophagy could antagonize the death-inducing effects of non-selective autophagy.
